# Ceramide galactosyltransferase (UGT8) is a molecular marker of breast cancer malignancy and lung metastases

**DOI:** 10.1038/sj.bjc.6605750

**Published:** 2010-07-20

**Authors:** P Dzieȩgiel, T Owczarek, E Plaz`uk, A Gomułkiewicz, M Majchrzak, M Podhorska-Okołów, K Driouch, R Lidereau, M Ugorski

**Affiliations:** 1 Department of Histology and Embryology, Medical University, T. Chałubińskiego 6a, Wrocław 50–368, Poland; 2 Department of Biochemistry, Pharmacology and Toxicology, Faculty of Veterinary Medicine, University of Environmental and Life Sciences, C. Norwida 31, Wrocław 50–357, Poland; 3 Laboratory of Glycobiology and Cell Interactions, Ludwik Hirszfeld Institute of Immunology and Experimental Therapy, Polish Academy of Sciences, R. Weigla 12, Wrocław 53–114, Poland; 4 Department of Thoracic Surgery, Medical University, Grabiszyńska 105, Wrocław 53–430, Poland; 5 Oncogenetics laboratory/INSERM U 735, Saint-Cloud, France; 6 Department of Histology and Embryology, Medical University, Świeȩcickiego 6, Poznań 61–781, Poland; 7 Department of Pathology, Lower Silesian Oncology Center, Wrocław 53–413, Pl. Hirszfelda 12, Poland

**Keywords:** breast cancer, metastasis, UGT8, molecular marker, GalCer

## Abstract

**Background:**

It was shown recently on the level of gene expression that *UGT8*, coding UDP-galactose:ceramide galactosyltransferase, is one of six genes whose elevated expression correlated with a significantly increased the risk of lung metastases in breast cancer patients. In this study primary tumours and their lung metastases as well as breast cancer cell lines were analysed for UGT8 expression at the protein level.

**Methods:**

Expression of UGT8 in breast cancer tissue specimens and breast cancer cell lines was analysed using IHC, real-time PCR and Western blotting.

**Results:**

Comparison of the average values of the reaction intensities (IRS scale) showed a significant difference in UGT8 expression between (1) primary and metastatic tumours (Mann–Whitney *U*, *P*<0.05), (2) tumours of malignancy grades G3 and G2 (Mann–Whitney *U*, *P*<0.01) as well as G3 and G1 (Mann–Whitney *U*, *P*<0.001) and (3) node-positive and node-negative tumours (Mann–Whitney *U*, *P*<0.001). The predictive ability of increased expression of UGT8 was validated at the mRNA level in three independent cohorts of breast cancer patients (721). Similarly, breast cancer cell lines with the ‘luminal epithelial-like’ phenotype did not express or weakly expressed UGT8, in contrast to malignant, ‘mesenchymal-like,’ cells forming metastases in nude mice.

**Conclusion:**

Our data suggest that UGT8 is a significant index of tumour aggressiveness and a potential marker for the prognostic evaluation of lung metastases in breast cancer.

The endoplasmic reticulum-localised enzyme UDP-galactose:ceramide galactosyltransferase (UGT8, CGT, C. E. 2.4.2.62) (Schulte and Stoffel, 1993; Kapitonov and Yu, 1997; Sprong *et al*, 1998) is responsible for the synthesis of galactosylceramide (GalCer), which is the major glycosphingolipid of myelin produced by oligodendrocytes in the central nervous system (CNS) and Schwann cells in the peripheral nervous system (Marcus and Popko, 2002). The exact role of GalCer in myelin sheath development and function is poorly understood; however this glycolipid is well recognised as a specific marker for the differentiation of these cells (Pfeiffer *et al*, 1993). Studies using antibodies suggested that GalCer may participate in signal transduction by regulating the intracellular calcium level and in this way mediating cytoskeletal rearrangements (Dyer and Benjamins, 1990, 1991). On the basis of a knockout mice model lacking UGT8, it was proposed that GalCer, together with sulphatide, is involved in myelin function and stability but not in its biogenesis (Bosio *et al*, 1996; Coetzee *et al*, 1996; Dupree *et al*, 1999). In addition to myelin, GalCer was also found in normal kidney (Ariga *et al*, 1980; Kodama *et al*, 1982), small intestine and colon (Natomi *et al*, 1993), liver (Nilsson and Svennerholm, 1982), testis (Vos *et al*, 1994), and milk (Bouhours and Bouhours, 1979).

Since the pioneering work of Hakomori and Murakami (1968), it was firmly established that neoplastic transformation and tumour progression are almost invariably associated with changes in the expression profiles of surface glycosphingolipids (Hakomori, 1996). However, there is very little information available on GalCer expression in human tumours. Only in studies on molecular markers in human astrocytomas and oligodendrogliomas was it found that high amounts of GalCer were present more frequently in oligodendrogliomas than in astrocytomas (Sung *et al*, 1996; Popko *et al*, 2002). Interestingly, more is known about the expression of UGT8 in cancerous tissues. Transcriptome profiling of prostate cancer cell lines showed that cells with metastatic properties express a much higher level of UGT8 mRNA in comparison with non-metastatic cells (Oudes *et al*, 2005). Using the same approach, we recently showed that *UGT8* is one of six genes whose elevated expression correlated with a significantly increased risk of lung metastases in breast cancer patients (Landemaine *et al*, 2008). It was also found that elevated expression of *UGT8* in breast cancer was significantly associated with ER-negativity, and therefore with a more malignant phenotype (Yang *et al*, 2006; Ruckhäberle *et al*, 2008).

As all the available information on the presence of UGT8 in breast cancer tissues was obtained only at the level of mRNA expression, primary tumours of different malignancy grades and their lung metastases were analysed for UGT8 expression at the protein level. In addition, presence of UGT8 and GalCer was determined in breast cancer cell lines representing different tumour phenotypes.

## Materials and Methods

### Tissue specimens and cell lines

Tissue blocks from 10 patients with primary breast cancer (invasive ductal carcinoma, IDC) were obtained from the Department of Pathology, Lower Silesian Center of Oncology (Wrocław, Poland) (eight cases) and Department of Pathology, Centre René Huguenin (Saint-Cloud, France) (two cases). Their corresponding lung metastases were collected also as tissue blocks from the Department of Thoracic Surgery, Wrocław Medical University (Poland), and Department of Pathology, Centre René Huguenin (Saint-Cloud, France) (Table 1). Thirty tissue specimens were obtained from patients who underwent resections of primary IDC at the Lower Silesian Center of Oncology, Wrocław (Poland) (Table 1). For mRNA analysis they were frozen at −80°C and for immunohistochemical staining they were fixed in 10% neutral-buffered formalin and embedded in paraffin. Paraffin sections, mounted on Superfrost Plus slides (Menzel Glaser, Braunschweig, Germany), were dehydrated and stained with haematoxylin and eosin. Malignant tumours were graded according to the classification of Bloom–Richardson with the modification of Elston and Ellis (1991). The study was approved by the Bioethical Committee of the Wrocław Medical University (no. KB-87/2005).

Three breast tumour series, the ‘MSK’, ‘EMS’, and ‘NKI’ cohorts (van de Vijver *et al*, 2002; Wang *et al*, 2005; Minn *et al*, 2005a, 2005b, 2007), for which microarray data are freely available, were also included in this study.

The following breast cancer cell lines were used in this study: MCF7, T47D, SKBR-3, BT-474, MDA-MB-231 (Cell Lines Collection of the Ludwik Hirszfeld Institute of Immunology and Experimental Therapy, Wrocław, Poland), MCF10CA1a.cl1 (provided by Dr S Santner, Karmanos Cancer Institute, Detroit, USA) and BO2 as a derivative of the MDA MB 231 cell line (provided by Dr Philippe Clezardin, INSERM U664, France). The cells were cultured in *α*-minimum essential medium (*α*MEM) supplemented with 10% foetal calf serum (FCS; Invitrogen, Carlsbad, CA, USA), 2 mM L-glutamine, and antibiotics.

### Immunohistochemistry

For immunohistochemical staining, 4-*μ*m-thick paraffin sections were deparaffinised in xylene and gradually rehydrated using ethanol. Endogenous peroxidase activity was blocked by a 5-min exposure to 3% H_2_O_2_. The cultured cells in eight-well culture slides (Becton Dickinson, Franklin Lakes, NJ, USA) were fixed in 4% neutral-buffered formalin for 15 min. Antigen retrieval was performed by exposure of the tissue sections and breast cancer cell lines to boiling Antigen Retrieval Solution (Dako, Glostrup, Denmark) in a microwave oven (250 W) for 15 min. Rabbit polyclonal antibodies directed against UGT8 were purchased from Atlas Prestige Antibodies (Stockholm, Sweden). The antibodies were diluted with Background Reducing Antibody Diluent (Dako). The sections were incubated with primary antibodies for 1 h at room temperature. Goat secondary antibodies (EnVision/HRP; Dako) directed against rabbit immunoglobulins were bound to a dextran framework conjugated with peroxidase. The reaction was developed using 3,3′-diaminobenzidine tetrachloride (DAB). Primary Negative Control (Dako) was used as the negative control. All tissue sections were counterstained with Mayer's haematoxylin.

The obtained photomicrographs were subjected to computer-assisted image analysis using a computer coupled to an Olympus BX-41 light microscope (Olympus, Tokyo, Japan) using the AnalySis software (Olympus). The degree of UGT8 expression was ranked using the modified semi-quantitative Immunoreactive Remmele Score (IRS) according to Remmele and Stegner (1987). The method takes into account both the proportion of stained cells and the intensity of the reaction, while its final results represent the product of the two parameters, with values ranging from 0 to 12 points (no reaction=0 points, weak reaction=1–2 points, moderate reaction=3–4 points, intense reaction=6–12 points). The results were subjected to statistical analysis using the Statisitica 7.1 software (StatSoft, Kraków, Poland). When groups of data were compared, which failed to satisfy assumptions of the parametric test, the Mann–Whitney *U*-test, the non-parametric equivalent of Student's *t*-test, was used. For matched samples of primary tumours and their metastases, the Wilcoxon signed rank sum test, the non-parametric version of a paired-samples *t*-test, was used. Correlations were tested by Spearman's correlation analysis. Results were considered statistically significant with *P*<0.05 in all analyses. Survivals times were determined by the Kaplan–Meier method and significance of differences were determined by log-rank test.

### RT-PCR and real-time PCR

Total RNA was isolated from the tissue samples using the RNeasy Fibrous Tissue Mini Kit (Qiagen, Hilden, Germany) according to the manufacturer's instructions. The protocol included on-column DNAse digestion to remove the genomic DNA. First-strand cDNA was synthesised using the SuperScript III First-Strand Synthesis System (Invitrogen, Carlsbad, CA, USA). The relative amounts of UGT8 mRNA were determined by quantitative real-time PCR with an iQ5 Optical System and iQ SYBR Green Supermix (Bio-Rad, Hercules, CA, USA) according to the manufacturer's protocols. GAPDH was used as a reference gene. The primers used were: realUGT8f/realUGT8r for UGT8 and SGAPDH/ASGAPDH for GAPDH (Table 2). The reactions were performed under the following conditions: initial denaturation at 94°C for 120 s, followed by 35 cycles of denaturation at 94°C for 30 s, annealing at 58°C for 30 s, and elongation at 72°C for 60 s. The specificity of the PCR was determined by melt-curve analysis for each reaction.

### SDS-PAGE and Western blotting

Cell lysates were obtained by treating the cells with RIPA lysis buffer (50 mM Tris-HCl, pH 8.0, 150 mM NaCl, 0.1% SDS, 1% IGEPAL Ca-630, 0.5% sodium deoxycholate). Proteins were quantified using a bicinchonic acid protein assay kit (Sigma-Aldrich, St Louis, MO, USA) and subjected to electrophoresis on 8% SDS-PAGE gel according to Laemmli. After electrophoresis, the proteins were transferred to a nitrocellulose membrane (Bio-Rad). UGT8 was detected with rabbit polyclonal antibodies (Atlas Antibodies, Stockholm, Sweden) and horseradish peroxidase-conjugated donkey anti-rabbit immunoglobulins (Jackson ImmunoResearch, West Grove, PA, USA). The bound antibodies were visualised with the Lumi-Light^PLUS^ Luminal/Enhancer Solution and Lumi-Light^PLUS^ Stable Peroxidase Solution (Roche, Basel, Switzerland). The light-sensitive membrane was then developed by incubating with the Kodak Developer and Kodak Fixer according to the kit's protocol (Kodak, Rochester, NY, USA).

### Purification of neutral glycolipids and thin-layer chromatogram binding assay

Neutral glycolipids were purified as described previously (Ugorski *et al*, 1989). Cell pellets corresponding to 10^8^–10^9^ cells were extracted with chloroform/methanol/water, 20/10/1, 10/20/1, and 10/10/1 by volume. Combined extracts were subjected to mild alkaline hydrolysis with 0.2 M KOH in methanol and desalted on a column of Sephadex G-25 superfine (Pharmacia Biotech, Uppsala, Sweden). The gangliosides were separated from the neutral glycolipids on a DEAE-Sephadex A-25 column (Pharmacia Biotech). The neutral glycolipids were further purified after acetylation on a Florisil column (Merck, Darmstadt, Germany) and analysed by high-performance thin-layer chromatography (HP-TLC) on silica gel 60 HP-TLC plates (Merck) with a solvent system of 2-propanol/15 M ammonia solution/methyl acetate/water, 75/10/5/15 by volume. (Ogawa *et al*, 1988). The standard for GalCer was obtained from Sigma-Aldrich. GalCer was detected by a TLC binding assay with rabbit polyclonal antibodies directed against GalCer (Sigma-Aldrich) and AffiniPure goat anti-rabbit IgG (Jackson ImmunoResearch) (Magnani *et al*, 1982). The latter were labelled with K^125^I (PerkinElmer, Waltham, MA, USA) using Iodo-Beads Iodination Reagent (Pierce, Rockford, IL, USA) according to the manufacturer's instructions.

## Results

### Expression of UGT8 in primary breast cancer tumours and their metastases to the lung

**Figure 1:**
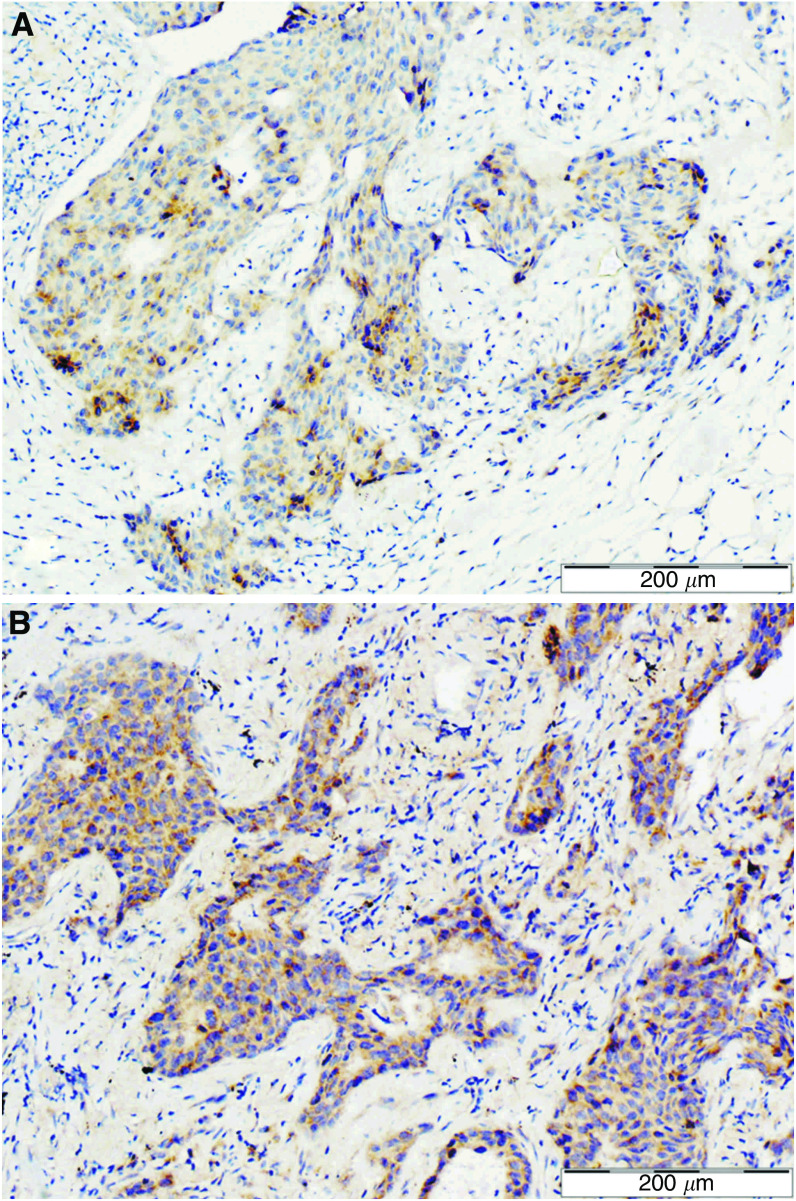
Immunohistochemical staining of primary breast carcinoma (**A**) and paired lung metastasis (**B**) with anti-UGT8 rabbit polyclonal antibodies.

**Figure 2:**
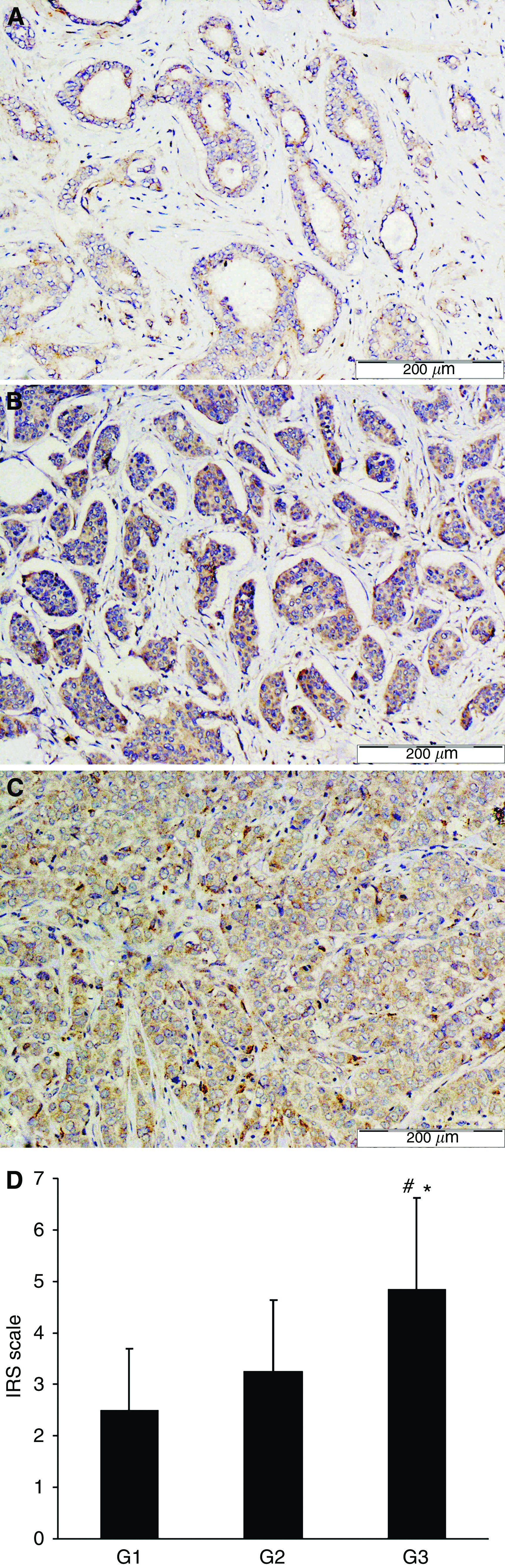
Immunohistochemical staining of primary breast carcinomas representing different malignancy grades (G1–G3). UGT8 expression in primary breast carcinomas of different malignancy grades: (**A**) G1 (*n*=9), (**B**) G2 (*n*=10), and (**C**) G3 (*n*=21). Reaction intensities with rabbit polyclonal antibodies for UGT8 (**D**) were calculated on the basis of the semi-quantitative IRS scale of Remmele and Stegner (1987) and are represented as means; ^*^P<0.01 for primary breast tumours of grade G3 as compared with primary breast tumour of grade G2, and ^#^*P*<0.001 for primary breast tumours of grade G3 as compared with primary breast tumours of grade G1 (Mann–Whitney *U*-test).

**Figure 3:**
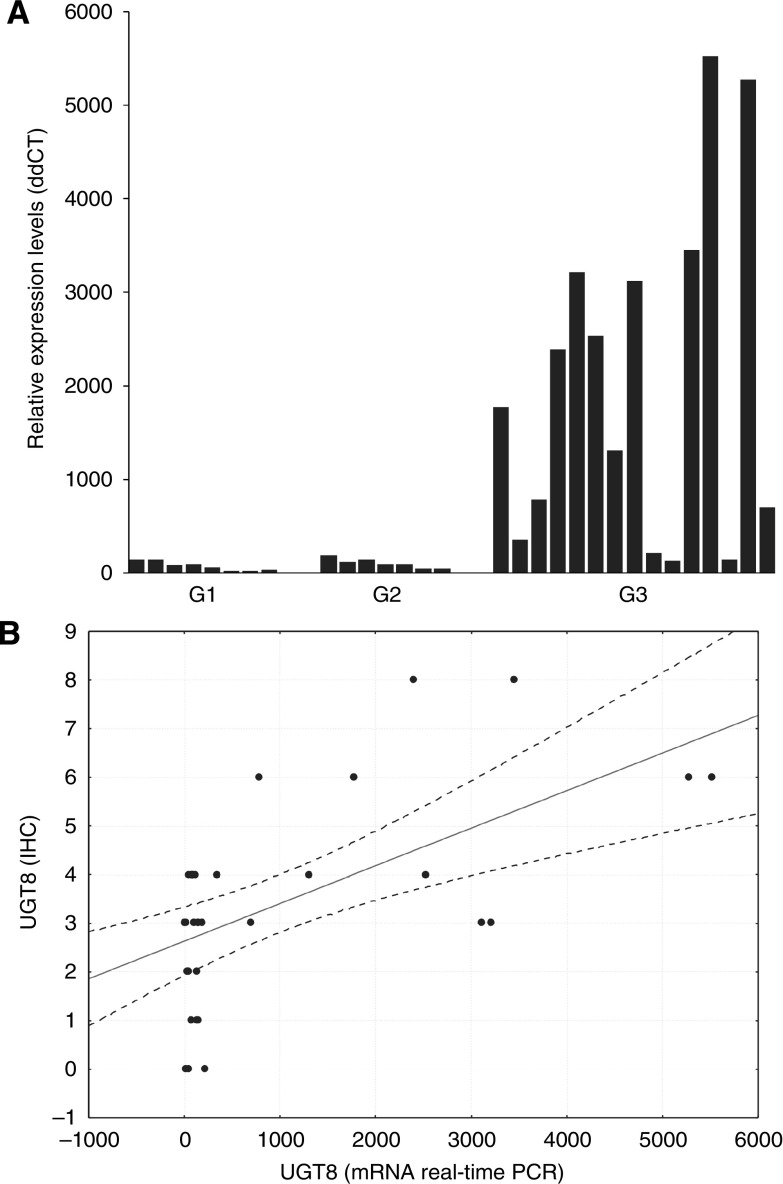
(**A**) Expression of UGT8 mRNA in primary breast tumours of different malignancy grades. Real-time RT-PCR was used to analyse UGT8 mRNA. UGT8 levels were normalised against GAPDH and cell line MCF-7 was assigned as a calibrator sample. (**B**) Positive correlation between intensity of UGT8 expression (immunohistochemistry, IHC) and UGT8 mRNA level (real-time PCR) in invasive ductal carcinoma. *r*=0.58, *P*<0.05 (Spearman's correlation).

**Figure 4:**
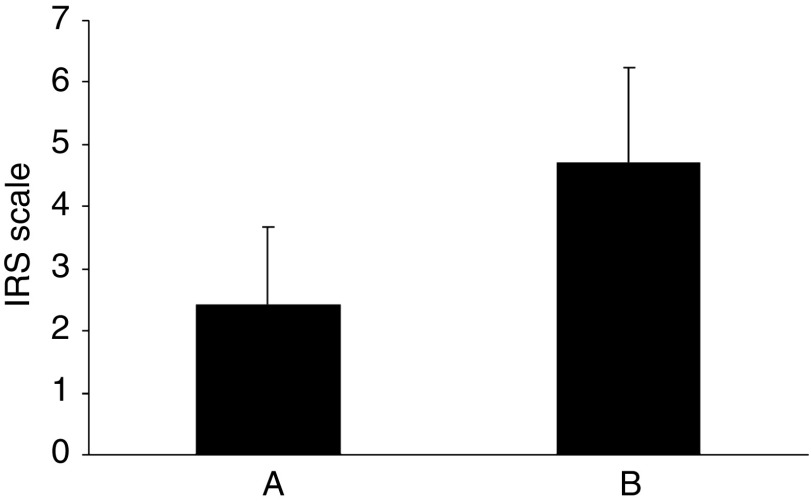
UGT8 expression (A) in node-negative invasive ductal carcinomas (*n*=22) and (B) node-positive invasive ductal carcinomas (*n*=18). *P*<0.05 for UGT8-expressing, node-negative primary breast tumours as compared with node-positive primary tumours (Mann–Whitney *U*-test). Reaction intensities with rabbit polyclonal antibodies for UGT8 were calculated on the basis of the semi-quantitative IRS scale of Remmele and Stegner (1987) and are represented as means.

On the basis of histological examination 10 primary breast carcinomas and their matched metastases were included in this study. The primary carcinomas were classified according to the Bloom–Richardson scale in the modification of Elston and Ellis (1991) as G1 (1 case), G2 (3 cases), and G3 (6 cases). Expression of UGT8 in the paraffin sections of the cancer tissue specimens was analysed using rabbit polyclonal antibodies. All primary tumours except one stained more weakly with anti-UGT8 antibodies than did the lung metastases (Figure 1). Comparison of the average values of the reaction intensities (IRS scale) showed a significant difference (Wilcoxon *t*-test, *P*<0.017) in UGT8 expression between primary and metastatic tumours (Table 3). Using IHC, UGT8 expression was also studied in the primary tumours according to their malignancy grades. For staining with anti-UGT8 antibodies, additional paraffin sections from 8 tumours of grade G1, seven tumours of grade G2, and 15 tumours of grade G3 were included. It was found that the expression of UGT8 in the tumour cells increased with increasing malignancy grade, reaching the highest values in the weakly differentiated cells (G3) (Figure 2A–C). When the average values of the reaction intensities (IRS scale) for tumours of different histological differentiation were compared by the Mann–Whitney *U*-test significant differences in UGT8 expression between malignancy grades G3 and G2 (*P*<0.01) as well as G3 and G1 (*P*<0.001) were found (Figure 2D). The results obtained at the protein level were confirmed when 30 primary tumours available as tissue specimens were also analysed by real-time PCR (Figure 3A). A significant positive correlation (*r*=0.58, *P*<0.05) between the expression of UGT8 protein and UGT8 mRNA was found using Spearman's correlation analysis (Figure 3B). The intensity of UGT8 staining in node-positive breast cancer tumours, according to the IRS scale, amounted on average to 4.7±1.53 and in node-negative tumours to 2.41±1.24 This difference also proved to be significant (*P*<0.001) (Figure 4).

**Figure 5:**
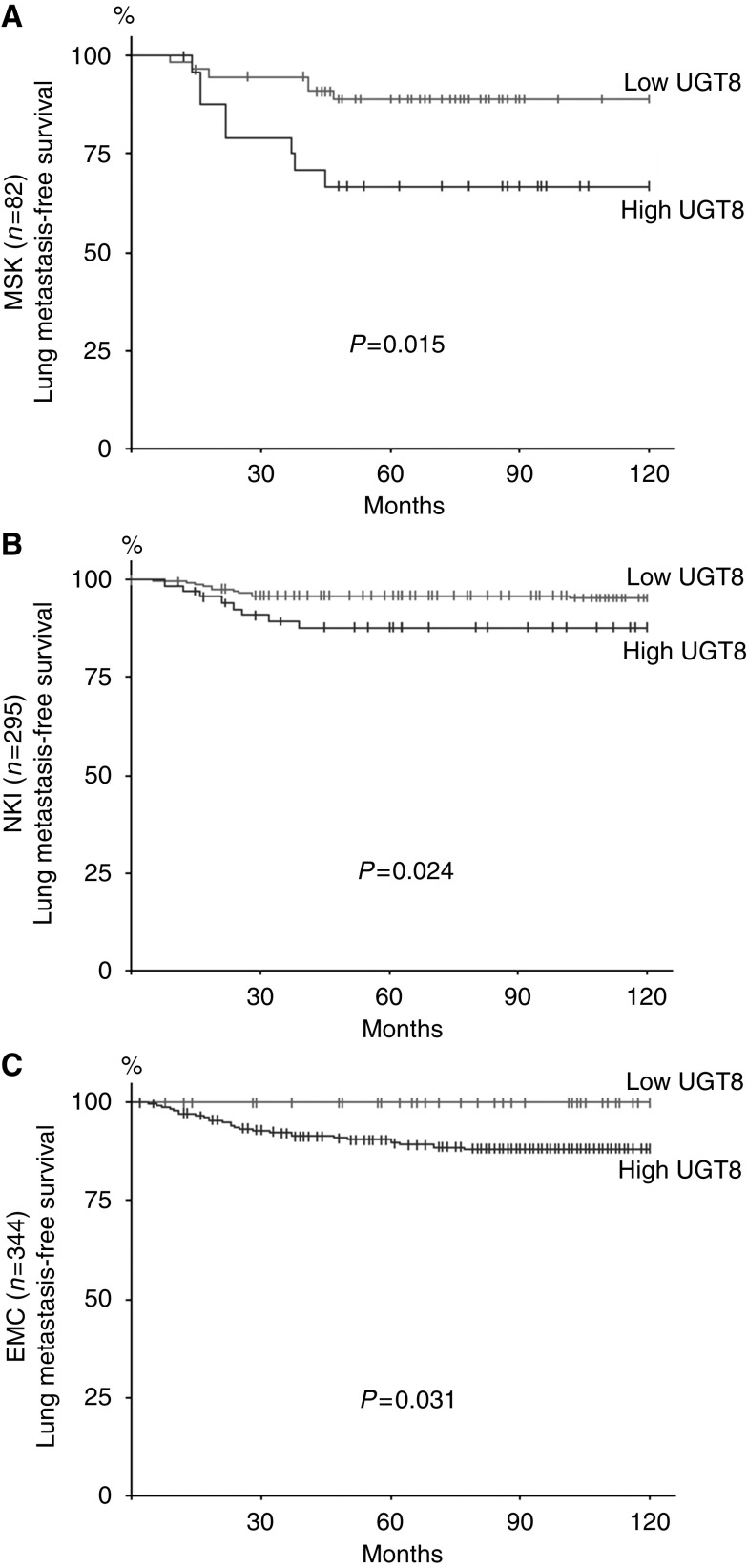
Validation of UGT8 expression as a predictive marker of lung metastasis in three independent series of breast cancer patients. Lung metastasis-free survival was analysed for (**A**) MSK (*n*=82), (**B**) NKI (*n*=295), and (**C**) EMC (*n*=344). Kaplan–Meier analysis distinguished patients who expressed high levels (high-risk group) and low levels (low-risk group) of UGT8. Patients with high expression of UGT8 had a shorter lung metastasis-free survival.

To validate the predictive ability of the elevated expression of UGT8 in primary breast tumours, the level of mRNA for UGT8 was analysed in three independent cohorts of breast cancer patients whose microarray data were available (van de Vijver *et al*, 2002; Wang *et al*, 2005; Minn *et al*, 2005a, 2005b, 2007) (Figure 5). In all three analysed cohorts, patients assigned to the high-risk group had significantly shorter lung metastasis-free survival.

### Expression of UGT8 in established breast cancer cell lines

**Figure 6:**
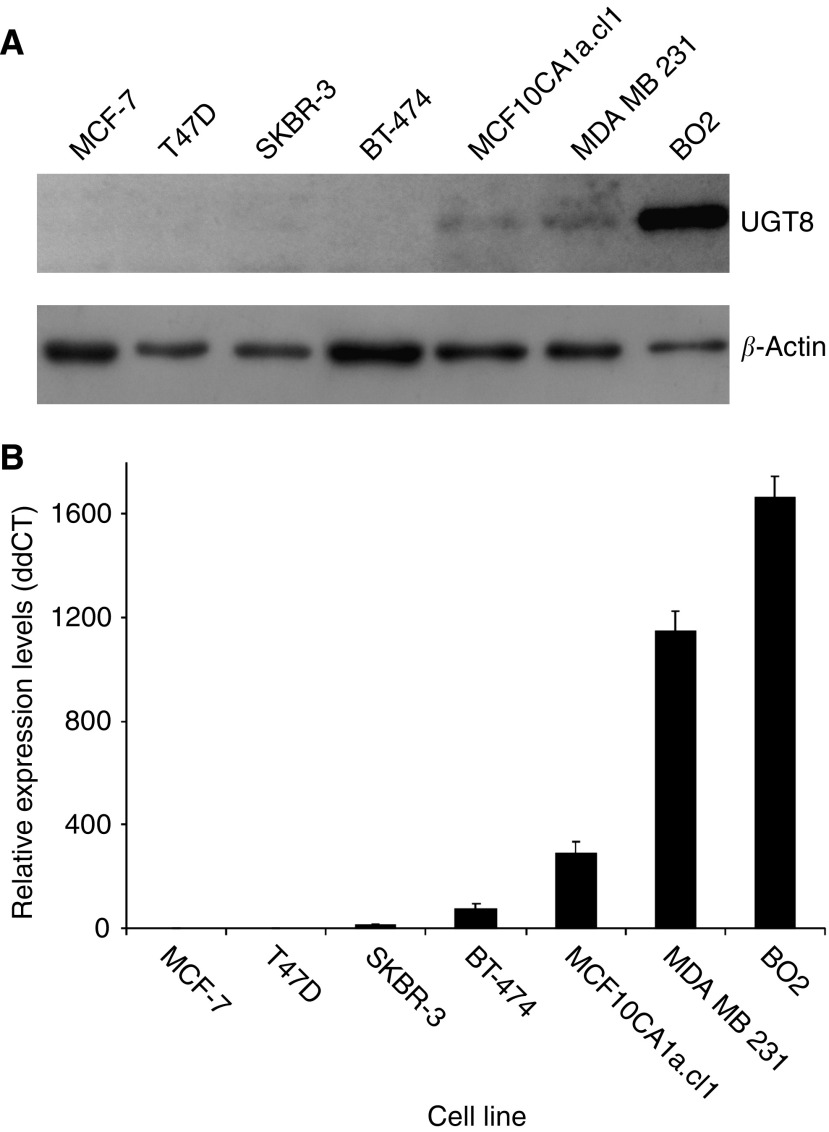
(**A**) Western blot analysis of anti-UGT8 rabbit polyclonal antibodies binding to cellular proteins of breast cancer cell lines. Cell lysates, equivalent to 15 *μ*g of protein, were separated by SDS-PAGE under reducing conditions on an 8% gel and electrophoretically transferred onto a nitrocellulose membrane. *β*-Actin served as an internal control. (**B**) Expression of UGT8 mRNA in breast cancer cell lines. Real-time RT-PCR was used to analyse UGT8 mRNA. UGT8 levels were normalised against GAPDH and cell line MCF-7 was assigned as a calibrator sample. Results are expressed as means.

The following breast cancer cell lines were used to analyse the expression of UGT8 with rabbit polyclonal antibodies: MCF-7, T47D, SKBR-3, BT-474, MCF10CA1a.cl1, MDA-MB-231, and BO2. Western blot analysis showed that high UGT8 expression was limited to the metastasising MCF10CA1a.cl1, MDA-MB-231, and BO2 cells (Figure 6A), and no binding was observed for the rest of the analysed cells. These results were confirmed by real-time PCR (Figure 6B) and immunohistochemical staining with anti-UGT8 polyclonal antibodies (data not shown).

### Detection of GalCer in established breast cancer cell lines

**Figure 7:**
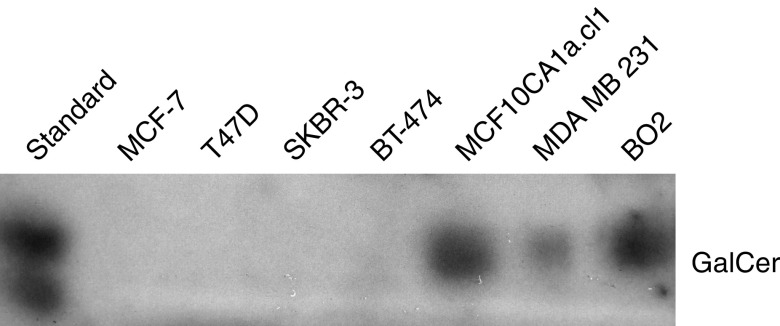
Immunostaining of neutral glycolipids from human breast cancer cell lines, separated by HP-TLC, with anti-GalCer rabbit polyclonal antibodies. For the analysed cell lines, an aliquot of total neutral glycolipids corresponding to 1 × 10^7^ cells was applied to the HP-TLC plate.

Neutral glycolipids purified from the breast cancer cell lines were separated on HP-TLC plates and immunostained with rabbit polyclonal antibodies directed against GalCer. Staining of GalCer, migrating as bands of appropriate mobility, was seen only in neutral glycolipid fractions isolated from metastasising breast cancer MCF10CA1a.cl1, MDA-MB-231, and BO2 cells (Figure 7). No bands were detected when using neutral glycolipids isolated from the rest of the analysed cell lines (BT474, SKBR-3, T47D, and MCF-7).

## Discussion

Breast carcinoma is the leading cause of mortality due to malignancy in Europe and North America. Death from breast cancer is mainly due to distant metastases, which are organ-specific and localise to bones, liver, lung, and brain (Fidler, 2003). On the basis of transcriptome analysis of primary tumours and distant metastases using genome-wide microarray techniques, it was proposed that the risk for developing metastases can be predicted by a ‘metastatic-gene signature’ expressed by subsets of primary tumours (van’t Veer *et al*, 2002; Ramaswamy *et al*, 2003; Weigelt *et al*, 2003; Driouch *et al*, 2007). With the use of a nude mice model it was further shown that clones of MDA-MB-231 breast cancer cells developing organ-specific metastases in bone or lung are characterised by the expression of specific subsets of non-overlapping genes (Kang *et al*, 2003; Minn *et al*, 2005a, 2005b). This finding suggested the existence of specific gene signatures determining the localisation of metastases in specific organs, which was confirmed in three independent series of breast tumours showing that the ‘lung metastasis signature’ is predictive of high risk for the development of lung metastases (Minn *et al*, 2005a, 2005b, 2007). Recently, a six-gene signature predicting breast cancer lung metastases obtained for metastatic human tissue specimens was published (Landemaine *et al*, 2008), which correlated with increased risk of lung metastasis in a series of 72 lymph node-negative breast tumours. These data were further validated on a larger series of samples (Driouch *et al*, 2009).

Among six genes highly overexpressed in lung metastases as compared with that in other breast cancer metastases, *UGT8*, which encodes an enzyme responsible for the synthesis of galactosylceramide, was found (Landemaine *et al*, 2008). In other studies on breast cancer, it was shown that elevated expression of *UGT8* was significantly associated with ER-negativity and therefore with a more malignant phenotype (Yang *et al*, 2006; Ruckhäberle *et al*, 2008). These studies indicating an elevated level of UGT8 in more malignant breast cancer cells were performed only at the level of mRNA expression using microarray analysis and quantitative RT-PCR. In our study we evaluated UGT8 protein expression in primary breast cancer tumours and their matched lung metastases using IHC. Significantly stronger staining with rabbit polyclonal antibodies directed against UGT8 was observed in the specimens from lung metastases than in paired primary tumours, confirming earlier results obtained at the mRNA level. These data suggested that UGT8 is associated in some way with tumour progression, and that its elevated level could be important in the development of lung metastases (see below). Therefore, to study the changes in UGT8 expression during increasing malignancy of breast cancer cells in more detail, samples of breast tumours having different malignancy grades were analysed for the presence of UGT8 at the protein as well as mRNA level. It was found that the amounts of UGT8 protein and mRNA increased with tumour malignancy grades, and highly significant differences in UGT8 expression were found in G3 tumours *vs* G2 tumours. Interestingly, highly increased expression of UGT8 was also observed in primary node-positive tumours as compared with that in node-negative primary tumours. When the predictive value of UGT8 expression was further analysed at the mRNA level in primary tumours of the 721 breast cancer patients of the three independent cohorts, the patients assigned to the high-risk group had significantly shorter lung metastasis-free survival. Therefore, our data suggest that UGT8 is a significant index of tumour aggressiveness and potential marker for the prognostic evaluation of lung metastases in breast cancer.

According to Lacroix and Leclercq (2004) breast cancer cell lines can be classified into three groups on the basis of their phenotype and invasiveness. The first group, including BT-483, MCF-7, T-47D, and ZR-75 cells, was named ‘luminal epithelial-like’ because the cells highly express such genes as ER, *CDH1* (E-cadherin), *TJP1* (zonula occludens-1), and *DSP* (desmoplakin-I/II), typical of the epithelial phenotype of breast cells. All these cells are weakly invasive. The second group, called ‘weakly luminal epithelial-like’, represented by SKBR-3 cells and BT-474, is similar to the first group, expressing the same epithelial markers, although at lower levels. The cells belonging to the third group are quite different as they express proteins found in mesenchymal cells, for example vimentin, and are highly invasive *in vitro*. They were named ‘mesenchymal-like’ (‘stromal-like’) and are represented by MDA-MB-231 and MCF10CA1a.cl1 cells. As the first two groups probably correspond to tumours of grades G1 and G2, and the ‘mesenchymal-like’ group could represent G3 tumours, we analysed the expression of UGT8 in different breast cancer cell lines. Expression of UGT8 at the mRNA and protein level in the established breast cancer cell lines correlated well with the results obtained for the clinical samples. Cells with the ‘luminal epithelial-like’ phenotype (MCF-7, T47D, SKBR-3, and BT-474) did not express or weakly expressed UGT8, in contrast to the malignant, ‘mesenchymal-like’ cells (MCF10CA1a.cl1, MDA-MB-231, and BO2) forming metastases in the nude mice model.

UGT8 is responsible for the synthesis of galactosylceramide, which is the major glycosphingolipid of myelin in the CNS and peripheral nervous system (Marcus and Popko, 2002). There is very little information available on GalCer expression in human tumours, except for human astrocytomas and oligodendrogliomas (Sung *et al*, 1996; Popko *et al*, 2002). Very little is also known about the possible functions of GalCer in tumour cells, which is in striking contrast to glucosylceramide (GlcCer), the other simple glycosphingolipid consisting only of ceramide and glucose residue. It is widely accepted that GlcCer is a mitogenic molecule, as stimulation of its synthesis decreases the intracellular pool of ceramide, which has an important function in programmed cell death as a proapoptotic agent (Radin, 2001; Taha *et al*, 2006). Interestingly, several lines of evidence suggest that overexpression of glucosylceramide synthase and accumulation of GlcCer can lead to the development of drug resistance in cancer cells (Lavie *et al*, 1996; Okazaki *et al*, 1998; Radin, 2001). Therefore we analysed the presence of GalCer in breast cancer cells and found that the ‘mesenchymal-like’ cells MDA-MB-231, BO2, and MCF10CA1a.cl1, each forming metastases in nude mice, are the only cell lines synthesising this glycolipid. This finding is in agreement with the hypothesis of Beier and Gorogh (2005), who proposed that accumulation of GalCer in tumour cells inhibits apoptosis, which facilitates metastatic cells to survive in the hostile microenvironment of the target organ. However, further functional studies are necessary to confirm this hypothesis.

In summary, we have shown for the first time that (1) expression of UGT8 is higher in breast cancer metastases to the lung than in matched primary tumours and that increased amounts of this enzyme in cancerous tissue are associated with progression to a more malignant phenotype, and (2) expression of UGT8 and GalCer is limited only to breast cancer cell lines forming metastases in a nude mice model.

## Figures and Tables

**Table 1:** Patients and tumour characteristics

	**Tumours without lung metastases**		**Tumours with lung metastases**	
Mean age in years (range)	58.4 (32–80)		59.8 (43–71)	
**Parameters**	**Number**	**%**	**Number**	**%**
Invasive ductal carcinoma (IDC)	30	100.0	10	100.0
				
*Size*				
T1 (<2 cm)	16	53.3	3	30.0
T2 (2–5 cm)	11	36.7	4	40.0
T3 (>5 cm)	3	10.0	3	30.0
				
*Lymph nodes*				
Negative	22	73.3		
Positive	8	26.7	10	100.0
				
*Grade*				
G1	8	26.7	1	10.0
G2	7	23.3	3	30.0
G3	15	50.0	6	60.0
				
*ER*				
Positive	20	66.7	4	40.0
Negative	10	33.3	6	60.0
				
*PR*				
Positive	13	43.3	7	70.0
Negative	17	56.7	3	30.0
				
*HER2 by IHC*				
Positive	6	20.0	2	20.0
Negative	24	80.0	8	80.0

Abbreviations: ER=estrogen receptor; IHC=immunohistochemistry; PR=progesterone receptor.

**Table 2:** Oligonucleotide primers used in real-time PCR experiments

**Target sequence**	**Name**	**Primer location in human cDNA**	**Nucleotide sequence of primers (5′ → 3′)**	**Product size (bp)**
UGT8	RealUGT8f	834 → 854	ATGGGTAAATGGTGCTAATG	334
	RealUGT8r	1145←1167	TCTGGTCATAGTATCATAATGG	
GAPDH	SGAPDH	536 → 553	TCACTGCCACCCAGAAGA	399
	ASGAPDH	916←935	TACCAGGAAATGAGCTTGAC	

**Table 3:** Immunohistochemical expression of UGT8 in primary breast carcinomas and paired lung carcinomas^a^

	**IRS scale** a	
**Number**	**Primary tumour**	**Lung metastasis**
1	2	4
2	3	2
3	1	3
4	2	3
5	1	1
6	1	2
7	2	4
8	2	4
9	2	3
10	2	4
		
Mean	1.8	3.0
S.d.	0.63	1.05
		
*P*	0.017	

Abbreviation: IRS=Immunoreactive Remmele Score.

a Reaction intensities with rabbit polyclonal antibodies for UGT8 were calculated on the basis of the semi-quantitative IRS scale of Remmele and Stegner (1987) and are represented as means.

*P*<0.017 for UGT8-expressing primary breast tumours as compared with that for matched lung metastases (Wilcoxon signed rank sum test).
